# The presentation and treatment of Dupuytren’s disease in Dutch general practitioner care

**DOI:** 10.1093/fampra/cmae065

**Published:** 2024-11-20

**Authors:** Roel J M van Straalen, Dieuwke C Broekstra, Paul M N Werker, Michiel R de Boer

**Affiliations:** Department of Plastic Surgery, University of Groningen, University Medical Center Groningen, HPC BB81, P.O. box 30.001, 9700 RB Groningen, The Netherlands; Department of Plastic Surgery, University of Groningen, University Medical Center Groningen, HPC BB81, P.O. box 30.001, 9700 RB Groningen, The Netherlands; Department of Plastic Surgery, University of Groningen, University Medical Center Groningen, HPC BB81, P.O. box 30.001, 9700 RB Groningen, The Netherlands; Department of Primary- and Long-term Care, University of Groningen, University Medical Center Groningen, P.O.-box 196, 9700 AD Groningen, The Netherlands

**Keywords:** Dupuytren’s contracture, primary care, epidemiology, symptoms, treatment

## Abstract

**Background:**

When research and management of Dupuytren’s disease (DD) shift from symptom relief to preventing contractures, general practitioner (GP) care may become more central to treatment. However, the presentation and course of DD in GP care are underexplored and this has been recognized as a knowledge gap that hinders effective treatment decisions. This study is the first to map the trajectory of DD patients in GP care.

**Methods:**

Using electronic health records from Dutch general practices in a regional research network, we conducted a registration-based cohort study in a dynamic population. Descriptive statistics detailed patient demographics, number of contacts, and symptoms per contact. The time and number of contacts before diagnosis were also analysed. Sankey diagrams illustrated the relationship between management options and symptoms.

**Results:**

Over a 16-year period, 84% of patients with a DD diagnosis had visited their GP for this reason, with 73% only having one GP contact. The diagnosis was made at first contact for 93% of patients. Initial contacts often reported a lump (57.3%), but this symptom was less frequent in subsequent visits. ‘Daily life impairment’ increased after the first contact. The most common management options were referral to secondary care (37.7%) and watchful waiting (35.1%).

**Conclusion:**

The diagnosis and management of DD in GP care are in line with the current guidelines. Less than half of the DD patients were referred to secondary care during follow-up. This may give room for preventive treatment that limits progression. Future studies should focus on the accuracy of diagnosis and the feasibility of effective treatments in GP care.

**Key messages**

- Over a 16-year period, 84% of patients with a Dupuytren’s disease (DD) diagnosis had visited their general practitioner (GP) related to DD, with 73% having only one GP contact. The diagnosis was made at first contact for 93% of patients.
- The current management of DD in GP care has only a limited burden on the primary healthcare system.
- This study gives insight into the trajectory of patients with DD in GP care.
- Future studies should focus on the feasibility of effective treatments in GP care.

## Introduction

Dupuytren’s disease (DD) is a common fibromatosis of the hand. The first signs of DD are the appearance of small nodules or pits in the palm, which are often asymptomatic and overlooked by patients. Progression of the disease may lead to the formation of fibrotic cords that can contract the finger joints. These flexion contractures occur in a later stage of the disease and may lead to functional limitations that can interfere with daily activities [1, 2]. Where the initial thought was that DD was always progressive, it has more recently been shown that the rate of progression varies considerably [3]. A retrospective study showed that 50% of patients developed progressive flexion deformities during a 10-year follow-up [4]. Other prospective studies reported progression in 21%–50% of patients within 7 to 18 years [5, 6].

Currently, there is no cure for DD. Therapy often starts when symptoms affect daily functioning. The current treatment at that point is mainly surgery, performed in secondary care, and aimed at reducing contractures of the fingers [7]. Although this is effective, the disease often recurs, requiring (multiple) re-operations.

Because of the considerable recurrence incidence, repetitive surgical treatment, and associated complications, the focus of DD research and management is shifting toward the prevention of contractures. A variety of less-invasive treatment options have been studied, with the aim of developing successful non-surgical disease-controlling treatments [8, 9]. A recent randomized controlled trial on the effectiveness of repeated intranodular adalumimab injections showed promising results with respect to softening and size reduction of DD nodules [10]. Disease-controlling treatments are ideally administered to people with early DD symptoms, who show signs of progression. Especially in countries with so-called gatekeeping systems, these patients first present themselves in GP care. Because GP care is less expensive and more easily accessible than secondary, specialized care, preventive treatment services could shift towards primary care in the future.

The presentation and course of DD in GP care have seldomly been investigated. The limited knowledge about the effectiveness of patient management strategies in GP care has been recognized as an important knowledge gap, impairing adequate treatment decisions [11, 12]. Current directives for GPs are concise and only describe three reasons for referral: (i) functional impairment, (ii) rapid progression of disease, and (iii) pain [13]. As such, the treatment and referral policies of GPs are mainly consensus-based instead of evidence-based. So far, only incidence and prevalence rates of DD in GP care have been studied. We recently showed that DD is not a rare disease in GP care. With an overall prevalence of 1.99% in 2021 and an incidence of 1.72/1000 person years in 2019 [14], the incidence was comparable to appendicitis or diverticulosis (both 1.6/1000 person years) [15].

Information on the presentation, course, and treatment of patients with DD in GP care is still lacking. This hampers joint decision-making between patient and GP, as there is a lack of scientific knowledge to make an informed decision.

This study aims to map the GP care pathway of patients presenting to GPs with DD by answering the following research questions: (i) what is the trajectory of DD patients in terms of symptoms and duration from first presentation to diagnosis and further patient management, (ii) what is the time to diagnosis, and which additional investigations are used, and (iii) which treatments for DD are currently provided in GP care.

## Methods

### Study design

This is a registration-based cohort study in a dynamic population of patients from general practices in the northern part of the Netherlands, where GPs act as gatekeepers for all patients in the healthcare system.

### Study population and setting

Data was used from the 'Academisch Huisarts Ontwikkel Netwerk' (AHON) registration database of the northern region of the Netherlands. It comprises care data of 58 participating general practices including data from around 500 000 patient records, with 49.6% males. Starting with three practices as the Registration Network Groningen in 1989, this later expanded in 2017 to become the AHON registry. General practices provide patient data from 5 years prior to the start of the registration at AHON [16].

For the current study, data up to and including the 31 December 2021 was extracted. A patient’s registration period started from registration and ended either by the patient’s death, termination of registration (e.g. by leaving the practice), the end of data collection of the practice, or the end of the study period (31 December 2021).

Studies using the AHON database do not fall within the scope of the Medical Research Involving Human Subjects Act (WMO) according to the medical ethics assessment committee of the University Medical Center Groningen (UMCG) (RR number 202100077) and therefore require no further ethical approval. We did obtain approval from the scientific committee of the AHON (ID number 74).

### Participants

Participants for the current study included all the patients aged 18 years or older with the diagnosis DD, regardless of when the diagnosis was made. The diagnosis DD was defined as a contact with ICPC code L99.03 (Dupuytren’s contracture) or as a contact in which DD was mentioned in the free text of the contact. Diagnosis DD was extracted from the free texts via a text-mining algorithm, described previously [14]. We then manually evaluated each contact with the ICPC code L99.03 or the letters ‘Dup’ in the free text retrospectively for all included patients. Contacts included physical appointments and appointments by phone, as fewer physical consultations occurred during the COVID-19 pandemic. First, we determined whether a contact was an appointment for DD based on the free text. If untrue, the contact would not be evaluated further.

### Outcomes/Data extraction

For all patients determined as DD patients, the free text of their contact(s) was evaluated for the description of multiple variables (Supplementary Appendix 1). Symptoms such as lump, curvature, daily life impairment (DLI), itching, and pain were registered. We described these on a patient level per contact and not specified per hand or finger. Other variables included the patient’s request for help, additional examinations, patient management options, and time between contacts. Finally, we evaluated whether the diagnosis of DD was made or was suspected by the GP.

Manual scoring of each contact was performed by the first author. This was validated by a second author (DB) who manually scored a random sample of 2% of all contacts. Both authors scored the data independently from each other. Their scores were compared and an agreement of > 0.8 (Cohen’s Kappa) was found for all variables (Supplementary Appendix 2).

### Statistical analysis

We aimed to gain a comprehensive understanding of the distribution and characteristics of contacts, symptom profiles, diagnostic processes for DD, patient management options, and the referral timeline.

All analyses were descriptive, using numbers and percentages, or medians and interquartile ranges (IQRs) as applicable. First, we described baseline characteristics including the number of patients and contacts, age, sex, and follow-up time. Symptoms per contact, patient requests, and patient management options, including additional examinations, were also presented.

Next, we described the time and number of contacts needed before DD diagnosis. The time interval between contacts was calculated by subtracting the date of diagnosis from the date of first presentation. The same was done to calculate the time to referral.

We also examined patterns between patient management choices and reported symptoms within subsequent contacts. To visualize these patterns we created Sankey diagrams. Under the assumption that every patient with DD presented with at least a lump (so also in cases where no symptoms were registered), we additionally included the following symptoms: curvature of the finger(s), DLI, and pain (all: yes/no). We also included the two most commonly chosen patient management options for the first three contacts. We combined the other management options under the value ‘other’.

A sensitivity analysis was conducted including only patients whose initial contact occurred within the period spanning from 2017 to 2022. This period was chosen because, for this timeframe, we had data from 10 years prior (the database was established in 2012 and practices provide data up to 5 years prior). In this way, we increased the probability to only include true initial contacts for DD.

All statistical analyses were performed with R (version 4.0.5) using the packages tidyverse, networkD3, ggplot2, and data.table.

## Results

### Baseline characteristics

**Figure 1.**
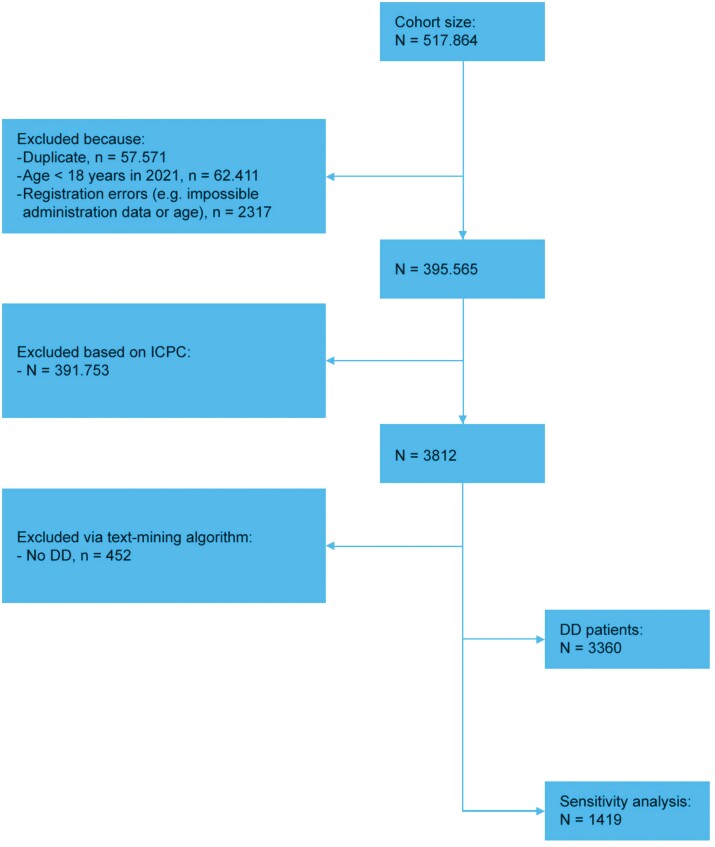
In-/Exclusion chart of registered persons and DD patients.

Within our cohort of 3360 patients with DD (Fig. 1), 2829 patients contacted the GP for DD (84.2%) for a total number of 4087 contacts for DD between 2004 and 2022. The sensitivity analysis included 1419 patients that had 1804 contacts.

The median age of the patients that contacted the GP was 70.3 years and 62% were male, compared to 70.3 years and 54.5% male in patients with DD who did not contact their GP during our study period. The median follow-up time was 5.2 (IQR 3–7.6) years after the first presentation. The first three contacts made up 94.93% of all contacts (Table 1).

**Table 1.** The number of contacts (%) per patient, presented for the complete DD population and for the subset of cases with the first contact between 2017 and 2022 (sensitivity analysis).

No. of contacts	Frequency (*n*), %	Sensitivity analysis Frequency (*n*), %
1 contact	2067 (73.1)	1155 (81.4)
2 contacts	488 (17.2)	189 (13.3)
3 contacts	162 (5.7)	49 (3.4)
4 contacts	69 (2.4)	19 (1.3)
5–10 contacts	40 (1.4)	6 (0.4)
>10 contacts	3 (0.1)	1 (0.1)

### Symptoms per contact

The symptom ‘lump’ was most often reported during the initial contact (57.3%, Table 2), but was less frequently reported in subsequent contacts. In contrast, the symptom ‘DLI’ shows an increase in frequency during subsequent contacts. Percentages for ‘curvature’ and ‘pain’ were relatively consistent across subsequent contacts, while ‘itching’ was rarely documented. The sensitivity analysis showed similar results, except for pain for which frequencies seem to increase with contacts.

**Table 2.** The number (%) of contacts with described symptoms presented for the complete DD population and for the subset of cases with the first contact between 2017 and 2022 (sensitivity analysis).

	Main analysis			Sensitivity analysis		
Symptoms	1st contact *N* = 2829, %	2nd contact *N* = 762, %	3rd contact *N* = 274, %	1st contact *N* = 1419, %	2nd contact *N* = 264, %	3rd contact *N* = 75, %
Lump	1622 (57.3)	285 (37.4)	66 (24.1)	886 (62.4)	101 (38.3)	19 (25.3)
Curvature	879 (31.1)	246 (32.3)	68 (24.8)	415 (30.3)	71 (26.9)	13 (30.7)
Daily life impairment	356 (12.6)	167 (21.9)	53 (19.3)	197 (13.9)	57 (21.6)	12 (16)
Pain	459 (16.2)	136 (17. 8)	47 (17.2)	252 (17.8)	58 (22)	23 (30.7)
Itching	7 (0.3)	1 (0.1)	1 (0.4)	3 (0.2)	1 (0.4)	1 (1.3)

### Patient requests

In 75.9% of all contacts, the patient requests were not registered. The most common registered patient requests were ‘whether they needed treatment or not’ (*n* = 540; 54.9%). Other patient requests were related to the diagnosis (*n* = 240; 24.4%) or related to concerns (*n* = 204; 20.7%).

### Patient management

**Figure 2.**
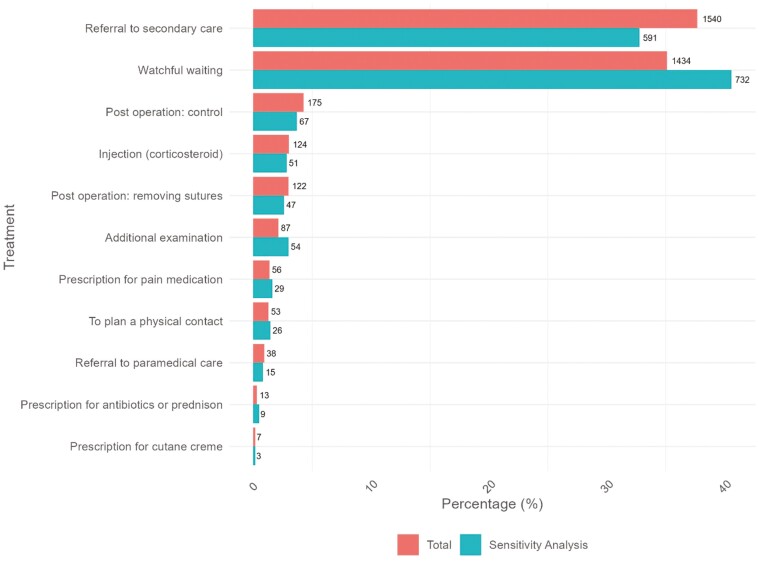
Registered management options in numbers and percentages.

Figure 2 shows the number and percentage of the recorded patient management options for all contacts. A total of 4138 management options were registered, including multiple management options within one contact (i.e. additional examination and prescription of painkillers). The most commonly registered management options were referral to secondary care (37.7%) and watchful waiting (35.1%). The sensitivity analysis showed watchful waiting (40.1%) as the most frequently recorded management option followed by referral to secondary care(32.8%).

### Additional investigation

Additional investigation was performed for 78 patients in 87 contacts (2.10%), compared to 46 patients in 53 contacts (2.9%) in the sensitivity analysis. Most additional investigations were initiated at the initial contact (75%). Radiographic (mostly ultrasound) and laboratory examination were the most frequently conducted examinations (*n *= 45, 45.9% and *n *= 38, 38.8%).

### Time to diagnosis

The diagnosis DD was made at first contact for 92% of all patients with anamnesis and physical examination. The diagnosis was made during a telephone consultation for 1% of the patients. For 183 patients (6.5%) DD was in the differential diagnosis at first contact, but the diagnosis was not (yet) confirmed. Additional investigation, such as X-ray, laboratory, or ultrasound examination, was conducted for 29 of these patients (16.9%).

Of the 183 patients with a delayed diagnosis, 51 patients returned for a second contact after a median time of 43 (IQR 9–343) days. At the second contact, 41 patients (80.4%) received the diagnosis DD. For one patient it took up to seven contacts over 3 years to come to the diagnosis of DD. The sensitivity analysis showed similar results (see Supplementary Appendix 3).

### Time to referral

As mentioned previously, in 37.7% of all contacts patients were referred to secondary care.

At the first presentation, 982 patients (34.7%) were immediately referred to secondary care. If referral did not occur at first contact, the overall median time to referral was 345 (IQR, 73–858) days. Patients presenting with a lump at first contact had a median time to the referral of 360 (IQR, 96–752) days, while patients presenting with contracture, pain, or DLI had a median time to the referral of 307 (IQR, 104–672), 210 (IQR 36–486), and 172 (IQR, 43–596) days.

Of patients who were managed differently (e.g. watchful waiting) at first presentation and saw the GP for a second time, 363 participants (47.6%) were referred to secondary care. The median time between the first presentation and the referral at the second presentation was 304 (IQR 97–749) days. In case patients were not referred at the second presentation, the referral was performed at the third presentation for 111 patients (40.5%) after a median time of 138 (IQR 32–483) days between the second and third contact. The sensitivity analysis showed a similar pattern of referrals at the first three contacts, although the proportions of referrals were somewhat lower overall (31.9%, 39%, and 28%, respectively).

### Symptoms and patient management

**Figure 3.**
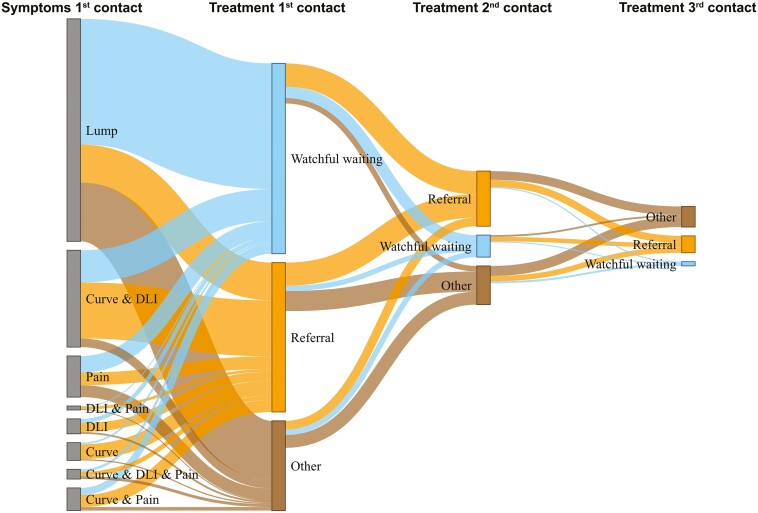
Sankey-flow diagram* of first three contacts. *The length of each ‘bar’ corresponds with the number of participants in each group and the width of each ‘flow’ represents the number of participants in this flow. The grey bars on the left represent the (combination of) symptoms at the initial contact. Note that the Sankey diagram illustrates the symptom ‘lump’, signifying the absence of descriptions for the symptoms curvature, DLI, or pain.

Figure 3 shows that most patients presented with only a lump at their first contact (*n* = 1466: 52%) for which the most frequent management was ‘watchful waiting’ (blue flow, first row, *n = *826, 56%). A smaller proportion of these patients were referred immediately to secondary care (orange flow, first row, *n *= 249: 17%). The most prevalent combination of symptoms at first presentation was the coexistence of finger curvature and DLI (*n *= 638: 23%). More than half of these patients were referred at the first presentation (orange flow, first row, *n *= 369: 58%).

Overall, the most frequently recorded patient management option at first contact was watchful waiting (blue bar, second column, *n* = 1248; 44%). Referral is the second most commonly chosen management at first contact (orange bar, second column, *n* = 984: 35%), predominantly for individuals with described curvature of the finger(s) (*n* = 968, 34%).

Referral was the overall most frequently recorded patient management option during the second contact (orange bar third column, *n = *363, 48%). Notably, after an initial watchful waiting management, referral became the predominant option at second contact (*n *= 153, 58%). Additionally, there was a relative increase in the proportion of ‘other’ patient management (*n *= 259, 34%) at the second contact, compared to the first contact (21%). ‘Other’ patient management included e.g. ‘post-operation control’ or ‘post-operation removal of sutures’. The sensitivity analysis produced similar findings (Supplementary Appendix 4).

We also examined and described the symptoms presented by patients during their second contact in Supplementary Appendix 5.

## Discussion

Our results show that 84% of patients with the diagnosis DD, registered by the GP, visited the GP over a period of 18 years, and 73% only had one GP contact for this diagnosis in this period. This suggests that the current management of DD in GP care has only a limited burden on the primary healthcare system.

The most commonly described symptom at first presentation is a lump, for which the predominant management is watchful waiting. Referral is the second most commonly reported patient management at first presentation, predominantly for individuals with described curvature of the finger. Diagnosis by anamnesis and physical examination was established at first contact for 93% of the participants and additional diagnostic investigations were rare. Sensitivity analyses including only patients whose initial contact occurred within the period from 2017 to 2022 (‘true initial contacts’) showed similar results.

We have not been able to locate similar work reporting the course of symptoms and treatments of patients with DD in GP care. It is important to note that our study specifically addressed the course of events in people with DD who actively seek medical assistance and explore their pathway in GP care, while other studies have reported on the progression of DD in the general population [5, 6]. Our registry data shows that less than half of the patients are eventually referred to secondary care. Part of this group could potentially benefit from early treatment with newly developed potentially disease-controlling agents, such as the intranodular adalimumab injection, thereby slowing down or stopping the progression of DD in the early phase of the disease. This would benefit the patients as well as society, as healthcare costs in primary care are usually lower than those in secondary care [17, 18]. As a first step towards such preventive treatment in GP care, future studies should examine the diagnostic accuracy of GP diagnosis against a gold standard. A second step would be to conduct feasibility research, as not every healthcare professional in GP care might be capable or willing to administer such a treatment. For instance, current guidelines for corticosteroid injections for trigger fingers recommend administration only by individuals with adequate proficiency and knowledge of anatomy, technique, and potential risks associated with the injection [13].

Our results show that the majority of the GPs adhered to the current national treatment guidelines for DD [13]. This guideline describes functional impairment, rapid disease progression, and pain as three criteria for referral. It is noteworthy that in 3% of cases, corticosteroid injections were recorded as a treatment for DD. One plausible reason for this treatment choice is that GPs may have still considered trigger fingers in their differential diagnosis. However, upon closer inspection of these contacts with corticosteroid injections as treatment, we observed that for 90.3% of the cases, the diagnosis DD was certain.

A Swedish cohort study previously reported the incidence of people seeking medical care for DD in primary, secondary, and tertiary care settings, along with the treatment trends observed in 2013 [19]. According to their findings, 1.6% of individuals received an unspecified injection. Assuming these were corticosteroid injections, our recorded rate of 3% is relatively high. However, direct comparison is not appropriate, as nearly all treatments in the Swedish study were administered in secondary or tertiary care.

Although rarely reported, some atypical treatments (e.g. prescriptions of antibiotics or prednisone) were registered. Detailed examination revealed that these were DD related, e.g. antibiotics were prescribed in case of wound infection after operation in secondary care.

When patients seek medical care for DD, the patient’s request should form the basis for the treatment. In our GP registry, the patient’s request was only reported in 24.1% of the contacts.

### Strengths and limitations

A strength of our study is the use of a large GP care registration-based dataset, that consisted of data of more than 3000 GP-registered patients. Another strength is that we manually evaluated the free text of each contact related to DD, creating a comprehensive description of what the GP registers.

Our study was conducted in the northern region of the Netherlands, in which healthcare is organized according to a gatekeeping system. Therefore, our results are more reflective of healthcare systems that also employ a gatekeeping approach. The gatekeeping approach is common in numerous European countries, Canada, Australia, and Brazil and is also in place in certain health plans in the USA. Noteworthy, however, is that the general population of the northern region of the Netherlands differs from the Dutch general population [20], which may make our results somewhat less generalizable for the whole country. However, there is no evidence to suggest that the pathway for DD patients differs from elsewhere.

A limitation in the use of registration-based data is that the primary goal of registration is to monitor patient care and not to collect data for research purposes. For example, the symptom ‘lump’ was less often reported than expected, which can presumably be attributed to the documentation of the GP who may not be documenting every physical aspect during each contact. Furthermore, in 75% of the cases, the patient’s request was not documented. This leads to potential under-registration of symptoms and patient requests related to DD, which may have biased our descriptions.

### Conclusion

This study shows that the diagnosis and management of DD in GP care are in line with the current guidelines. Less than half of the DD patients were referred to secondary care during follow-up, mainly because they presented with curvature, impairment, or pain. This study gives insight into the trajectory of patients with DD in GP care. With future disease-controlling treatment on the horizon, future studies should focus on the accuracy of diagnosis and the feasibility of effective treatments in GP care.

## Supplementary Material

cmae065_suppl_Supplementary_Appendix

## Data Availability

The datasets used and/or analysed during the current study are available from the corresponding author and AHON commission on reasonable request.
