# Supplementary material for: The presentation and treatment of Dupuytren’s disease in Dutch general practitioner care
Source: Fam Pract. 2024 Nov 20;42(2):cmae065. doi: 10.1093/fampra/cmae065 (PMC11811527; doi:10.1093/fampra/cmae065)
Supplement: cmae065_suppl_Supplementary_Appendix [file cmae065_suppl_supplementary_appendix.docx]

**Appendix**

Appendix 1: Method: Table Variables

| **Variable** | **Range of scores** |
| --- | --- |
| Lumb/nodule | 0 no  1 yes  2 yes/increased  9 No information |
| Contracture |  |
| Hinder in daily life |  |
| Itching |  |
| Pain |  |
| Patient concerns | 1 what is it?  2 treatment indication?  3 fear/concerns  9 No information |
| Diagnostic tests | 0 no  1 ultrasound  2 X-ray  3 laboratory research  4 CT  5 MRI  6 EMG  9 No information |
| Treatment | 0 wait and see  1 referral  2 Extra examination  3 injection  4 post surgery control  5 post surgery removal stitches  6 antibiotics  7 to plan physical consult  8 oral painkillers  9 No information |
| Diagnosis/suspicion | 0 No DD  1 Diagnosis  2 Differential diagnosis/suspicion  9 No information |
| Secondary diagnosis? | 0 no  1 yes  9 No information |

Appendix 2: Method: Outcomes/Data extraction

| **Agreement** | **Cohen’s Kappa** | **Percentage agreement** |
| --- | --- | --- |
| Contact DD (yes/no): | 0.88 | 94% |
| Ledderhose | NA | 100% |
| Lump | 0.93 | 97% |
| Contracture | 0.98 | 99% |
| Daily Life Impairment | 0.82 | 94% |
| Itching | NA | 100% |
| Pain | 0.89 | 97% |
| Patient Request | 0.89 | 98% |
| Diagnostic Tests | 0.83 | 94% |
| Treatment | 0.93 | 95% |
| Diagnosis/Suspicion | 0.94 | 98% |
| Secondary reason DD | 0.91 | 97% |
| *Dupuytren’s disease (DD), Not Applicable (NA)* | | |

Appendix 3: Results: Time to diagnosis, sensitivity analysis

The diagnosis DD was made at first contact for 91.8% of the participants. For 112 participants (7.9%) DD was in the differential diagnosis at first contact, but the diagnosis was not (yet) confirmed. Of these 112 participants, 27 participants returned for a second contact after a median time of 36 days. At second contact 21 participants (78%) received the diagnosis DD. Of the remaining unclear diagnosis only 3 returned for a third contact after a median time of 36 days, in which all 3 participants (100%) received the diagnosis.

Appendix 4. Results: Sensitivity analysis Sankey Diagrams

*
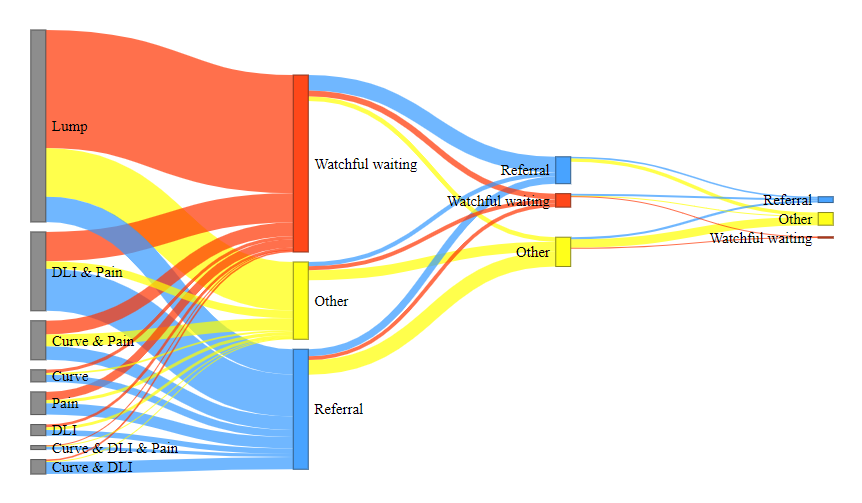
*

*Figure 4. Sankey-flow diagram of first three contacts (sensitivity analysis).*

Figure 4 visualises the symptoms presented by participants at first contact for the sensitivity analysis. Most participants presented with a lump at the initial contact (n = 728, 51%) for which the most frequent treatment was 'watchful waiting’ (red arrow, 1st flow, n = 448, 62%). A smaller proportion of these participants received direct referral to secondary care (blue arrow, 1st flow, n = 96, 13%).

The most prevalent *combination* of symptoms at first presentation was the coexistence of pain and DLI (n = 299, 21%). More than half of these patients were referred at first presentation (blue arrow, 1st flow, n = 159, 53%).

Overall, the most frequently recorded treatment option at first contact was watchful waiting (red bar 2nd column, n = 671, 47%). The treatment option referral was the second most commonly chosen treatment at first contact (blue bar, 2nd column, n = 455, 32%), predominantly for individuals with described pain or curvature of the finger(s).

Other was the overall most frequently recorded treatment option during the second contact (yellow bar 3rd column, n =111, 42%). This increased proportion of 'other' treatments at second contact, compared to the first contact, was most seen following referral at first contact. This might be attributed to including treatments such as ‘post-operation: control’ or ‘post-operation: removing sutures’.

Notably, after an initial treatment of watchful waiting, referral became the predominant chosen treatment at second contact (n = 59, 60%).

During the third contact, 'other' treatments were more prevalent (yellow bar 4th column, n = 48, 64%) than watchful waiting or referral.

Appendix 5. Results: Sankey diagrams for 2^nd^ contacts including those for the sensitivity analyses

*
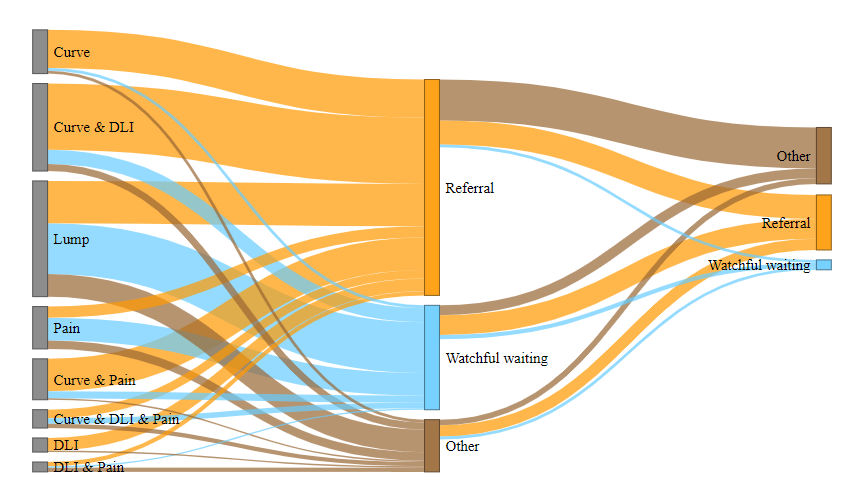
*

*Figure 5. Sankey-flow diagram of second and third contact after* ***Watchful waiting at first contact.***

*
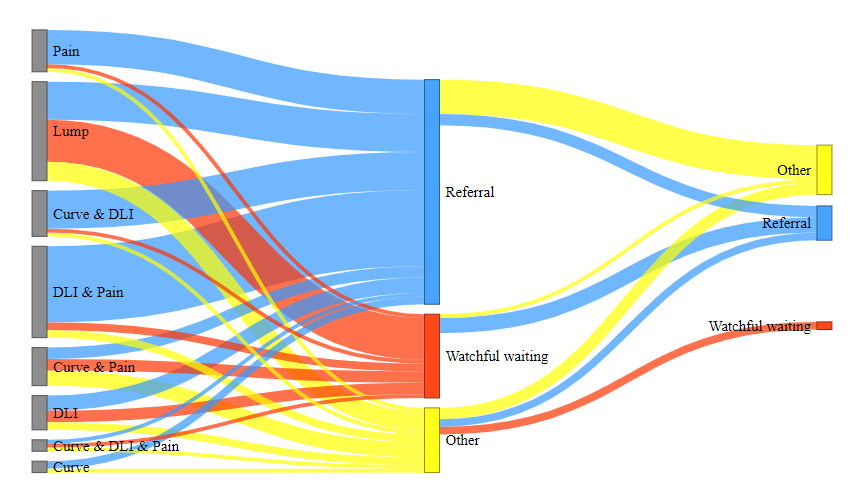
*

*Figure 6. Sankey-flow diagram of second and third contact after* ***Watchful waiting at first contact (sensitivity analysis).***

Figure 5 visualises the symptoms presented by participants during their second contact after watchful waiting as the initial contact. Compared to the first contact, it shows an increase in the proportion of symptom combinations involving curvature, DLI and pain (n = 182, 69%) (see figure 3, n = 1363, 48%). The most chosen treatment at second contact was referral (n=153, 58%).

We also see that after referral at second contact, other treatments (e.g. post-operation control) became the most prevalent at third contact. And after watchful waiting at second contact, referral became the most chosen treatment at third contact. As shown in figure 6 the sensitivity analysis showed similar results.


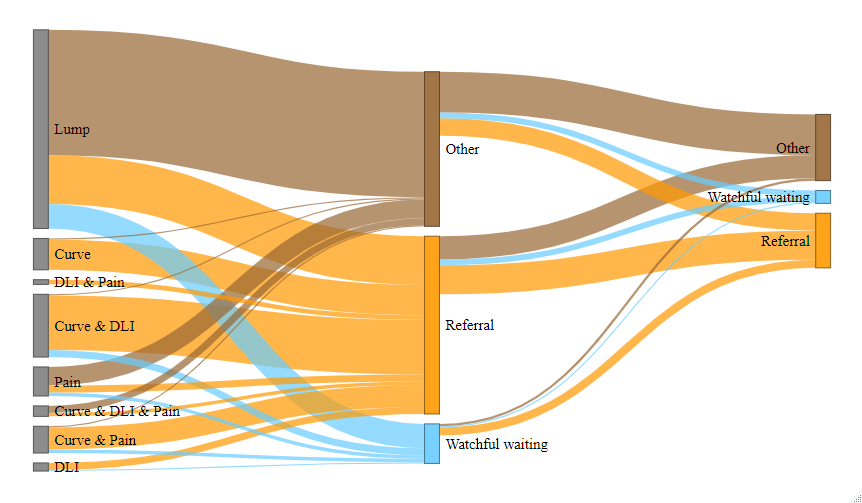


*Figure 7. Sankey-flow diagram of second and third contact after* ***Referral in first contact.***

*
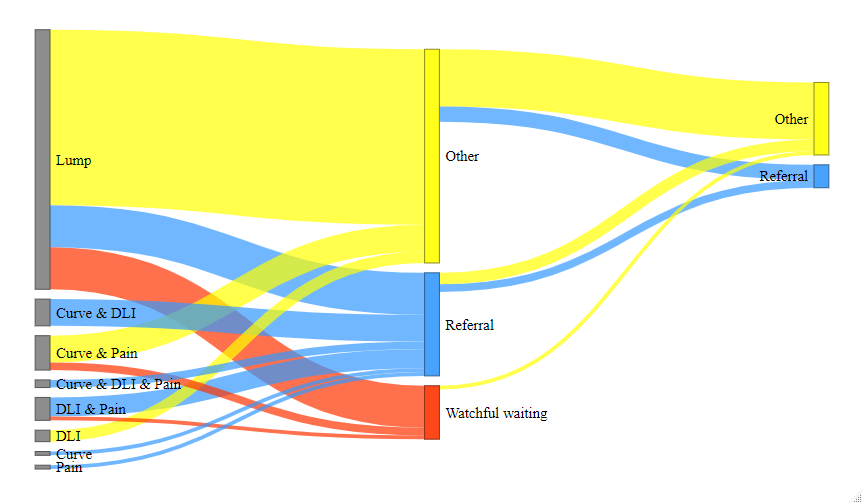
*

*Figure 8. Sankey-flow diagram of second and third contact after* ***Referral in first contact (sensitivity analysis).***

Figure 7 shows the symptoms presented by participants during their second contact after referral treatment at the initial contact.

We see a higher proportion of participants presenting a lump (n = 171, 53%), compared to the symptom (combinations) presented after watchful waiting as an initial treatment (see figure 5, n=82, 31%). Important to note here is that the symptom lump is a group made under the assumption that every patient with DD presented with at least a lump (so also in cases where no symptoms were registered).

We also observed an increase in proportion of ‘other’ treatments (n= 133, 42%) compared to treatments at first contact (n=597, 21%). Referral is the overall most common chosen treatment at second contact (n= 153, 48%).

The sensitivity analysis (figure 8) shows that other treatments are most prevalent at second contact, especially for the symptom lump. Also noteworthy is that when curvature is described, referral remains the most frequently chosen treatment.


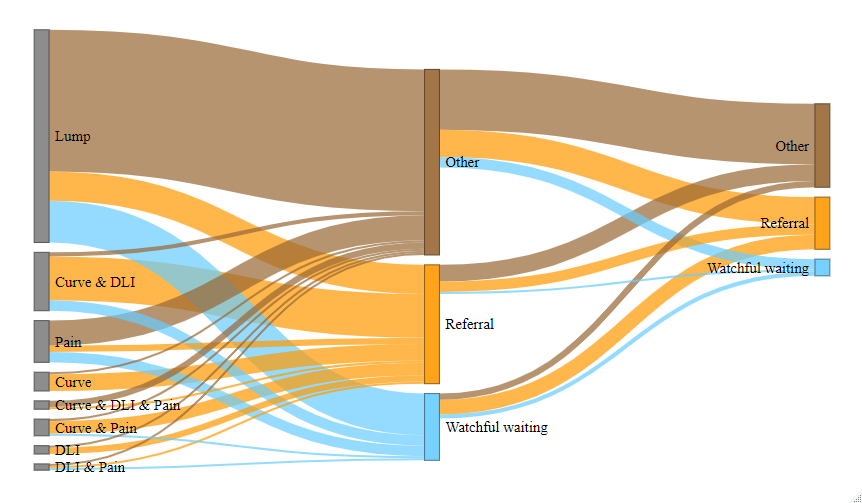


*Figure 9. Sankey-flow diagram of second and third contact after* ***Other in first contact.***


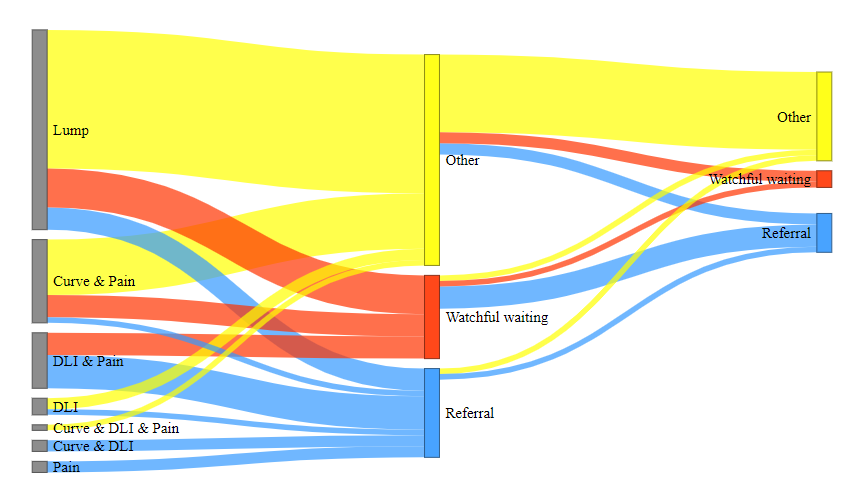


*Figure 10. Sankey-flow diagram of second and third contact after* ***Other in first contact (sensitivity analysis).***

Figure 9 shows the symptoms presented by participants during their second contact after other treatments at the initial contact.

We again see a high proportion of participants presenting a lump (n = 102, 57%). Other treatments (n= 89, 50%) remain the most chosen treatment at second contact. Referral is the second most common chosen treatment at second contact (n= 57, 32%). The sensitivity analysis (figure 10) shows similar results.
